# Prévalence des cervicalgies et douleurs des membres supérieures chez les utilisateurs d'ordinateur en milieu professionnel à Casablanca (Maroc)

**DOI:** 10.11604/pamj.2013.14.157.2329

**Published:** 2013-04-24

**Authors:** Nawal Seknaji, Wafaa Rachidi, Samira Hassoune, Saadia Janani, Samira Nani, Abderrahmane Maaroufi, Ouafaa Mkinsi

**Affiliations:** 1 Service de rhumatologie, CHU Ibn Rochd de Casablanca, Maroc; 2 Laboratoire d'Epidémiologie, Faculté de Médecine et de Pharmacie de Casablanca, Maroc

**Keywords:** Cervicalgies, douleurs, conditions ergonomiques, facteurs psychosociaux, neck pain, pain, ergonomic conditions, psychosocial factors

## Abstract

**Introduction:**

Les cervicalgies et douleurs du membre supérieur (CDMS) affectent des millions d'utilisateurs d'ordinateurs dans les pays développés. L'objectif de ce travail était de déterminer la prévalence des cervicalgies et douleurs du membre supérieur (CDMS) à Casablanca et identifier les différents facteurs de risque qui y sont impliqués, afin de définir les sous-groupes à haut risque, et d’établir des plans d'intervention efficaces.

**Méthodes:**

Il s'agit d'une étude transversale, menée chez 118 employés de bureau francophones et utilisant l'ordinateur, à Casablanca. Les données ont été collectées de Décembre 2011 à Juin 2012, en utilisant la version française d'un questionnaire validé: Maastricht-Upper-extremity-Questionnaire.

**Résultats:**

118 utilisateurs d'ordinateur en milieu professionnel ont été recrutés. Parmi les employés enquêtés, 93% ont rapporté des CDMS localisées au moins à un site. Les plaintes les plus fréquemment rapportées étaient les douleurs des épaules et les cervicalgies (48,3% et 46,6% respectivement). Nous n'avons pas remarqué de différence significative des fréquences des douleurs selon le genre. Nos résultats concordent avec les données de la littérature où il existe un certain consensus quant à l'impact des mauvaises conditions ergonomiques sur les troubles musculo-squelettiques du membre supérieur en milieu professionnel. De même, les études récentes examinant les effets combinés et/ou interactifs tant des facteurs biomécaniques/physiques que de facteurs psychosociaux professionnels vont de pair avec nos résultats.

**Conclusion:**

Il en ressort que les stratégies d'intervention visant à réduire les CDMS doivent agir essentiellement sur 3 volets, le premier correspond à l'amélioration et la sensibilisation quant au respect des conditions ergonomiques, le second concerne l'amélioration des conditions psychosociales. Enfin le troisième volet vise la rationalisant de la demande de travail.

## Introduction

Les cervicalgies et douleurs du membre supérieur (CDMS) affectent des millions d'utilisateurs d'ordinateurs dans les pays développés [1]. C'est ainsi qu'une définition universelle des CDMS fut élaborée par la méthode DELPHI, afin d'unifier le langage et permettre une comparaison entre les différentes études. Elle définit les CDMS par l'ensemble des plaintes au niveau de la région cervicale et du membre supérieur, d'origine non traumatique et non causées par une maladie systémique [2].

Dans les Pays-Bas, où la population active est estimée à 7 millions d'habitants, le coût annuel de ces troubles musculo-squelettiques est estimés à 2.1 milliards d′Euros annuellement [3]. Le Maroc est l'un des pays en voie de développement où les systèmes informatiques sont largement utilisés, surtout en secteur libéral, afin de supporter un développement industriel rapide. Néanmoins, les publications concernant les CDMS en rapport avec l'utilisation d'ordinateurs y sont limitées. Le but de ce travail était de déterminer la prévalence de ces douleurs et identifier les différents facteurs de risque aussi bien physiques que psychosociaux impliqués dans les cervicalgies et douleurs du membre supérieur, afin de définir les sous-groupes à haut risque, et d’établir des plans d'intervention précis et efficaces.

## Méthodes

**Figure 1:**
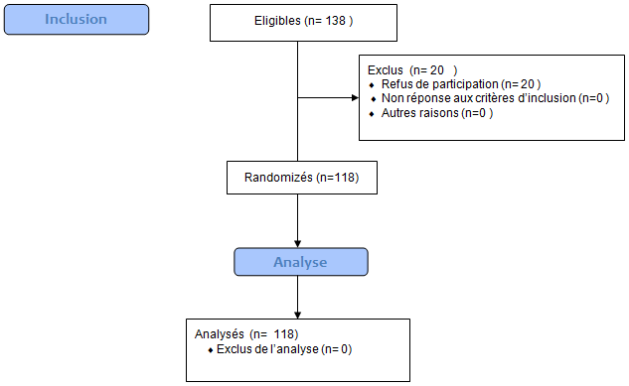
Diagramme CONSORT

Il s'agit d'une étude transversale, menée chez 118 employés de bureau francophones et utilisant l'ordinateur, à Casablanca. Ont été inclus dans cette étude les employés de bureau occupant le même poste durant au moins 06 mois et utilisant l'ordinateur pendant au moins deux heures par jour. Les critères d'exclusion étaient la présence de maladies affectant le système ostéo-articulaire et musculaire comme les rhumatismes inflammatoires, d'autres connectivites, et l'existence d'antécédent de chirurgie de la région cervicale et/ou du membre supérieur. La population étudiée représentait surtout le secteur libéral, où l'usage des systèmes informatiques est important. Avant leur inclusion dans l′étude, les participants ont été informés des objectifs de l′enquête et leur consentement oral a été obtenu avant l′administration du questionnaire. Les 20 personnes ayant refusé de participer ont été exclues de l’étude (Figure 1). Par ailleurs, l'anonymat et la confidentialité des données ont été respectés. L'accord du comité d’éthique n'a pas été nécessaire car ce dernier ne donne son avis que sur les études interventionnelles.

Les données ont été collectées de Décembre 2011 à Juin 2012, en utilisant la version française d'un questionnaire validé: Maastricht-Upper-extremity-Questionnaire, élaboré en 1999, et dont la traduction en langue française s'est faite par 2 traducteurs différents. Il contient 95 questions groupées en 11 items, auxquels la réponse s'effectue durant environ 30 min. Le premier item évalue les caractères démographiques des employés (âge, sexe, ancienneté, etc..), les 7 items suivants portent sur les principaux facteurs de risque potentiellement impliqués (1.le poste de travail, 2.la position du corps, 3. le contrôle du travail, 4.les demandes de travail, 5.la durée et qualité des pauses, 6.le support social et 7.environnement du travail). Les 3 items suivants évaluent le siège des douleurs, leurs caractères ainsi que leurs conséquences.

Nous avons calculé la prévalence des cervicalgies et des douleurs du membre supérieur (épaules, bras, coudes, avant-bras poignets et mains), ainsi que leurs intervalles de confiance à 95%. Les participants qui ont rapporté des DCMS ont été ensuite scindés en 2 groupes: Les cas modérés à sévères ont été définis par la persistance de la douleur pendant plus qu'un mois, l’évolution vers une faiblesse du membre supérieur, l'existence d'une douleur continue et permanente, et la perte d'un travail à cause des douleurs des CDMS. Les cas de faible sévérité ont été définis ainsi par l'absence de ces caractéristiques.

Les associations entre les variables qualitatives continues ont été étudiées en utilisant le test chi deux. Nous avons considéré la valeur P ≤ 0.05 comme statistiquement significative. Les analyses statistiques ont été réalisées en utilisant le logiciel SPSS version 16.0.

## Résultats

### Les caractéristiques démographiques et professionnelles

Nous avons recruté 118 utilisateurs d'ordinateur en milieu professionnel. Les hommes représentaient 55% de la population étudiée. 41,5% de la population avaient un âge inférieur à 29 ans. En ce qui concerne l'ancienneté, 64,6% des hommes et 50,9% des femmes ont déclaré occuper le même poste durant moins de 5 ans. Concernant la durée d'exposition, 54,2% des sujets passaient 6 à 8 heures/jour devant l'ordinateur, alors que 13,6% ont déclaré passer 9 heures ou plus (Tableau 1). Vingt-huit pour cent des personnes ont déclaré avoir des postes de travail non-conformes aux critères de l'OSHA (l′Agence Européenne pour la Santé et la Sécurité au Travail). A part l'incapacité à garder une bonne position durant le travail, et l'incapacité à garder les épaules soulevées pendant plus de 2 heures par jour, nettement plus fréquente chez les femmes (p=0,001 et p=0,007 respectivement), nous n'avons pas noté de différence entre les 2 sexes quant à l'exposition aux autres facteurs de risques physiques et psychosociaux.

**Tableau 1:** Caractères démographiques des utilisateurs d'ordinateur en milieu professionnel à Casablanca- 2012

	Total n(%)	Hommes n(%)	Femmes n(%)
**Age**			
≤29	40 (41,5)	28 (43,1)	21 (39,6)
30 - 39	32 (27,1)	21 (32,3)	11 (20,8)
≥ 40	37 (31,4)	16 (24,6)	21 (39,6)
**Ancienneté**			
1 an - 5 ans	69 (58,5)	42 (64,6)	27 (50,9)
6 ans - 10 ans	19 (16,1)	7 (10,8)	12 (22,6)
≥11 ans	30 (25,4)	16 (24,6)	14 (26,4)
**Heure de travail en PC / jour**			
2 à 5 heures	38 (32,2)	22(33,8)	16(30,2)
6 à 8 heures	64 (54,2)	34(52,3)	30(56,6)
≥ 9 heures	16 (13,6)	9(13,8)	7(13,2)

### La prévalence des CDMS

Parmi les employés enquêtés, 93% ont rapporté des CDMS localisées au moins à un site. La prévalence des CDMS, selon leur localisation, ainsi que leurs intervalles de confiance 95%, sont présentées dans le Tableau 2. Parmi les sujets qui souffraient de douleurs, 29% étaient des cas modérés à sévères, dont 65,6% étaient des femmes. 56,3% se plaignaient de douleurs dans plus d'une seule localisation. 2,72% accusaient des douleurs du membre supérieur en totalité. Les plaintes les plus fréquemment rapportées sont les douleurs des épaules et les cervicalgies (48,3% et 46,6% respectivement), suivies par les douleurs du poignet, du bras, des mains et des coudes (26,3%, 24,6%, 21,2% et 12,7% respectivement). Les douleurs de l'avant-bras étaient les moins fréquentes (9,3%). Nous n'avons pas remarqué de différence significative des fréquences des douleurs selon le sexe (Tableau 2).

**Tableau 2:** Prévalence des CDMS selon le genre chez les utilisateurs d'ordinateur en milieu professionnel à Casablanca- 2012

Siège de la douleur	nombre de sujets atteints	Prévalence globale (IC95%) (n=118)	Prévalence chez les hommes (IC95%) (n=65)	Prévalence chez les femmes (IC 95%) (n=53)	P*
Cou	55	46.6 [37.6; 55.6]	46.2 [34.1; 58.3]	47.2 [33.8; 60.6]	1
épaules	57	48.3 [39.3; 57.3]	52.3 [40.2; 64.4]	43.4 [30.1; 56.7]	0,36
Bras	29	24.6 [16.8; 32.4]	26.2 [15.5; 36.9]	22.6 [11.3; 33.9]	0,6
coude	15	12.7 [6.7; 18.7]	10.8 [3.3; 18.3]	15.1 [5.5; 24.7]	0,5
Avant-bras	11	9.3 [4.1; 14.5]	10.8 [3.3; 18.3]	7.5 [0.41; 14.6]	0,77
poignets	31	26.3 [18.4; 34.2]	24.6 [14.1; 35.1]	28.3 [16.2; 40.4]	0,67
mains	27	21.2 [13.8; 8.6]	21.5 [11.5; 31.5]	[9.9; 3.17]	1

* P: degré de signification

### Les facteurs de risque

D'après l'analyse bi variée, le seul facteur de risque associé aux cervicalgies était la non possibilité d'ajuster la hauteur de la chaise (p=0,02). (Tableau 3). Les facteurs de risque des douleurs des épaules étaient l’âge (p=0,035), la persistance de l'employé dans une position inconfortable (p=0,02), le fait d'effectuer des tâches non variées pendant le travail (p=0,003), et le caractère non amical des superviseurs (p=0,03). Il n'existe aucune corrélation entre les douleurs des épaules et les conditions du poste du travail (hauteur du bureau et de la chaise, position de l’écran et du clavier, etc..).

**Tableau 3:** Facteurs de risque d'ordre physiques et démographiques des CDMS selon les localisations- Casablanca, 2012

Localisations des douleurs	Facteurs de risque	Analyse bi variée	
		Khi deux	p
Cervicalgies	Je peux ajuster la hauteur de ma chaise	5,39	0,02
Douleurs de l’épaule	L’âge	6,7	0,035
	Pendant mon travail je suis assis dans une position inconfortable	5,2	0,022
	Je décide mes propres changements de tâche	5,4	0,02
	Dans mon travail j'effectue des tâches différentes	9,1	0,003
Douleurs du coude	Quand j'utilise la souris ma main est placée au même niveau que mon avant-bras	4	0,04
	Je travaille des heures supplémentaires pour finir mon travail	4,1	0,04
	L'air à l'intérieur de mon bureau est trop froid	4,4	0,03
Douleurs de l'avant-bras	Je trouve difficile de finir mes tâches à l'heure	4,7	0,03
	Mon atmosphère de travail est confortable.	8,5	0,003
Douleurs des mains	L’âge	7,09	0,028
	Je trouve difficile de finir mes tâches à l'heure	4,6	0,03
	Je prends des heures supplémentaires pour finir mes tâches de travail	4,9	0,025
	Je n'ai jamais assez de temps pour finir ma tâche de travail.	4,2	0,04
	J'exécute des tâches sans ordinateur	4	0,04

Concernant les douleurs des bras, les principaux facteurs de risques identifiés étaient le non développement des compétences au travail (p=0,033), l'absence de créativité au travail (p=0,05), et l'incapacité à organiser les horaires des pauses (p=0,005). Parmi les facteurs de risque identifiés pour les douleurs du coude on note comme facteurs physiques; le fait que la main ne soit pas placée au même niveau que l'avant-bras durant l'utilisation de la souris (p=0,04), et la présence d'un air froid à l'intérieur du bureau (p=0,03). Les facteurs psychosociaux significativement associés étaient l'importance des tâches de travail, la difficulté de les finir à l'heure (p=0,04), et la non créativité au travail (p=0,03) (Tableau 4). Concernant les douleurs de l'avant-bras, L'analyse par test khi deux a révélé comme principal facteur de risque physique le travail dans une atmosphère non confortable (p=0,003).

**Tableau 4:** Facteurs de risque psychosociaux des CDMS selon les localisations. Casablanca, 2012

Localisations des douleurs	Facteurs de risque	Analyse bi variée	
		Khi deux	p
Douleurs de l’épaule	L’âge	6,7	0,035
	Je décide mes propres changements de tâche	5,4	0,02
	Mes superviseurs sont amicaux	4,4	0,03
Douleurs des bras	Dans mon travail j'apprends de nouvelles choses	4,5	0, 033
	Je dois être créatif dans mon travail	3,67	0,05
	Je peux décider quand faire une pause	7,8	0,005
	Je dois être créatif dans mon travail	4,2	0,03
Douleurs de l'avant-bras	Dans mon travail j'apprends de nouvelles choses	6,1	0,013
	Je dois être créatif dans mon travail.	4,2	0,03
	Je trouve mon environnement de travail bon	7,5	0,006
	Le déroulement des opérations se fait sans conflits	14,67	≤0,001
Douleurs du poignet	Mon travail développe mes capacités	3,6	0,05
	Dans mon travail j'apprends de nouvelles choses	6,5	0,01
Douleurs des mains	Mon travail développe mes capacités	7,1	0,007
	Dans mon travail j'apprends de nouvelles choses.	15	0,00
	Je dois être créatif dans mon travail.	9,25	0,002

Les facteurs de risque psychiques identifiés sont l'absence de créativité au cours du travail (p=0,03), l'absence d'apprentissage de nouvelles compétences dans le travail (p=0,013), le déroulement des opérations dans un environnement conflictuel (p≤0,0001), l'incapacité à avoir des renseignements liés au travail (p=0,007), et la difficulté de finir ses tâches à l'heure (p=0,03). Les douleurs du poignet étaient significativement associés au non apprentissage de nouvelles choses au cours du travail (p=0,01). Les principaux facteurs de risque impliqués dans les douleurs des mains étaient l’âge avancé (p=0,028), la non-exécution de tâches sans ordinateurs (p=0, 04), la difficulté de finir ses tâches à l'heure (p=0,03), et la nécessité de travailler des heures supplémentaires pour accomplir son travail (p=0,025).

Parmi les facteurs psychiques, nous avons décelé l'absence de créativité au travail (p=0,002), et le non apprentissage de choses nouvelles au cours du travail (p=0,002) (Tableau 4)

### Les conséquences des douleurs

Comme conséquences, nous avons noté une gêne de l'activité professionnelle chez 25,5% des sujets, une faiblesse du membre supérieur et une gêne des loisirs chez 18,2%. La durée moyenne de gêne des activités quotidiennes par les douleurs était de 12 jours, avec des extrêmes de 2 jours à 140 jours. Parmi les sujets qui souffrent de CDMS, 12,7% se sont absentés du travail, 17,8% ont déclaré avoir consulté chez un médecin, 37% ont eu recours aux médicaments, 9% ont bénéficié d'une kinésithérapie et 11% ont eu recours à d'autres méthodes (ostéopathie, acupuncture, etc..). Par ailleurs, 2,7% des sujets ont déclaré avoir utilisé des dispositifs pour soulager la douleur (minerves, attelles, etc..).

## Discussion

Il s'agit de la première étude marocaine évaluant la prévalence des cervicalgies et douleurs du membre supérieur chez les utilisateurs d'ordinateurs au bureau. Parmi les employés enquêtés, 93% ont déclaré avoir des douleurs dans au moins une localisation au membre supérieur durant plus d'une semaine. Cette prévalence dépasse de loin celles observées aussi bien dans les pays développés, tel que les Pays-Bas (28%) [3], que dans les pays en voie de développement: Sri-Lanka (56,9%), Grèce (54%) et Soudan (70%) [4–6]. Contrairement aux résultats d'une étude Danoise similaire où les cas modérés à sévères représentent moins de 3% de la population, dans notre étude, 29% des cas étaient considérés modérés à sévères.

Les prévalences des cervicalgies (46,6%) et des douleurs des épaules (48,3%) étaient de loin les plus élevées. Ceci concorde avec les résultats retrouvés en Allemagne [7] ainsi qu'aux USA où une étude réalisée par Gerr et col a retrouvé une prévalence des cervicalgies et douleurs de l’épaule de 46% VS 34% pour les douleurs du bras, avant-bras et poignet [8]. Contrairement à l’étude de Ranasinghe, [4] ayant démontré une prédominance nettement supérieure des douleurs du bras, avant-bras et poignet chez la population Sri-lankaise.

Aux USA, les CDMS coutent à l'industrie environ 45 à 54 millions de dollars annuellement, par le biais de l'absentéisme et des dépenses médicales qui y sont liées [3]. Dans notre étude nous n'avons pas calculé le cout médical exact des CDMS, toutefois les dépenses directes et indirectes seraient considérables puisque 17,8% des sujets ont déclaré avoir consulté chez un médecin à cause des CDMS, et 45,7% des sujet sont déclaré s’être absenté du travail au moins une fois durant l'année précédente, la durée moyenne d'absentéisme étant de 13 jours.

En dehors des conséquences matérielles, les CDMS semblent avoir un impact considérable sur la qualité de vie puisque 18,2% des sujets ont déclaré avoir des répercussions sur leurs activités de loisirs VS 15,4% chez la population Sri Lankaise [4].

A l'exception des douleurs de l’épaule, légèrement plus fréquentes chez les hommes (52,3% vs 43,4%) et les douleurs du coude, plus importantes chez les femmes (15,1% VS 10,8%), nous n'avons pas noté de prédominance selon le sexe pour les autres localisations (cou, bras, avant-bras, poignets et mains).

Ceci diffère des résultats de l’étude soudanaise de Shahla, et celle danoise menée par Anderson et col qui a montré une prévalence des cervicalgies et douleurs des épaules nettement supérieure chez les femmes que les hommes [9]. L’étude de Lassen et col a également montré une prévalence des cervicalgies chez 42% des utilisateurs d'ordinateurs de sexe féminin vs 24% chez ceux de sexe masculin [7]. Dans la population grecque, les douleurs du poignet et des mains sont significativement plus fréquentes chez les femmes que les hommes [10].

Ces différences de prévalence selon le sexe ont été expliquées par des différences d'exposition aux facteurs de risque physiques et psychosociaux liés au travail [7, 10]. Néanmoins dans la population marocaine, à part la position au travail et le fait de garder ses épaules soulevées durant plus de 2 heures, il ne semble pas y avoir de différence entre les 2 sexes quant à l'exposition aux différents facteurs de risque. Par ailleurs, nous n'avons pas identifié le sexe comme facteur de risque pour les CDMS.

Dans notre étude, l’âge avancé a été identifié comme facteur de risque des scapulalgies, et des douleurs des mains, ceci pourrait s'expliquer par la fréquence accrue avec l’âge des lésions dégénératives tendineuses au niveau de l’épaule, et arthrosiques digitales.

Notre étude a retrouvé comme principal facteur de risque des cervicalgies la non possibilité d'ajuster sa hauteur de chaise. En effet, le travail dans un poste non-conforme implique des positions vicieuses du corps (cou en flexion etc. ') d'où une instabilité et une souffrance des éléments musculo-squelettiques [3, 11, 12]. Selon Ortiz-Ernandez [13], la persistance pendant une longue durée dans une position vicieuse (cou et dos courbé) augmente la pression intra discale, d'où la mise en tension des ligaments jaunes et les douleurs musculaires.

Pour les autres localisations, les principaux facteurs de risque ergonomiques identifiés dans notre étude étaient la position inconfortable pour les scapulalgies, l'utilisation inadéquate de la souris de l'ordinateur pour les douleurs du coude, et le mauvais emplacement de l’écran de l'ordinateur et du clavier pour les douleurs de l'avant- bras, poignet et des mains. Ceci concorde avec les données de la littérature scientifique où il existe un certain consensus quant à l'impact des mauvaises conditions ergonomiques sur les troubles musculo-squelettiques du membre supérieur en milieu professionnel [7, 14, 15]. Par ailleurs, plusieurs publications dont celles de Bongers [3] et Smideley [16] ont identifié comme principaux facteurs de risque des plaintes actuelles l'existence d'antécédents de douleurs cervicales et du membre supérieur.

Concernant les facteurs environnementaux, la présence d'un air froid au bureau a été principalement impliquée dans les douleurs du coude, alors que le travail dans un environnement bruyant était étrangement associé aux douleurs du poignet.

Plusieurs études dont celles de Bongers, Hannan, Shahla et Hoogendoorn [1, 7, 17, 18], ont identifié comme principaux facteurs étiologiques psychosociaux une demande accrue de travail, l'absence de support social de la part des collègues et superviseurs, la non-participation à la prise des décisions, et l'insatisfaction de son travail.

Dans notre étude, à l'exception des cervicalgies, les facteurs étiologiques d'ordre psychosociaux ont été identifiés pour toutes les localisations des douleurs au membre supérieur. En plus de ceux identifiés dans la littérature, elles comportent le déroulement des opérations dans un environnement conflictuel, le non développement des capacités et l'absence de créativité dans le travail, l'incapacité à décider des horaires des pauses et l'accès difficile à l'information.

Ces découvertes vont de pair avec les études récentes examinant les effets combinés et/ou interactifs tant des facteurs biomécaniques/physiques que de facteurs psychosociaux professionnels [19, 20]. L′interaction entre les facteurs psychosociaux d'agression, les demandes accrus de travail, les expositions ergonomiques et la réponse individuelle complexe à ces facteurs définissent un nouveau model appelé: le style de travail d'une personne.

Ce modèle de style de travail est basé sur l′hypothèse selon laquelle la manière dont un individu exécute ses tâches de travail, en réaction à une demande accrue de travail, au sein de facteurs d'agression psychosociaux, et dans de mauvaises conditions ergonomiques, peuvent augmenter le risque de développement des plaintes du membre supérieur ou entretenir des symptômes préexistants [21, 22]. Bien que notre travail n'ait pas étudié le modèle de style de travail, il en a évalué différents composants tel le rôle d'une demande accrue de travail, et la réponse comportementale à ces demandes qui peuvent exposer ces travailleurs aux facteurs tant biomécaniques que psychosociaux.

## Conclusion

De façon générale, les résultats de notre étude concordent avec ceux de la littérature. Les cervicalgies et les scapulalgies sont de loin les localisations les plus fréquentes des CDMS chez les utilisateurs d'ordinateurs en milieu professionnel. Leurs facteurs étiologiques regroupent d'abord les facteurs physiques liés au non-respect des bonnes conditions ergonomiques de travail tant de la part des employés que des employeurs, les facteurs environnementaux, ainsi que des agressions psychosociales au lieu de travail. Au sein de ce modèle étiologique, une demande accrue de travail semble jouer un rôle central prépondérant en favorisant la surexposition à l'ensemble des facteurs. Ces résultats suggèrent que les stratégies d'intervention visant à réduire les CDMS doivent agir essentiellement sur 3 volets, le premier correspond à l'amélioration et la sensibilisation des employés et employeurs quant au respect des conditions ergonomiques (création de postes de travail conformes aux critères de l'agence Européenne pour la Santé et la Sécurité au Travail), le second concerne l'amélioration des conditions psychosociales (demande du travail, sa difficulté, la dépendance d'autres collègues et le support social des collègues et superviseurs). Enfin le troisième volet vise la limitation de la surexposition aux différents facteurs de risque en rationalisant la demande de travail.
